# Concomitant Coronary Artery Disease and Persistent Left Superior Vena Cava in a Patient Presenting With Symptomatic Sinus Bradycardia: A Case Report

**DOI:** 10.7759/cureus.37579

**Published:** 2023-04-14

**Authors:** Muneeb Jan, Usha Kumari, Zair Hassan, Nikhil R Daggula, Salim Surani

**Affiliations:** 1 Internal Medicine, Rehman Medical Institute, Peshawar, PAK; 2 Internal Medicine, Khyber Teaching Hospital, Peshawar, PAK; 3 Medicine, Dow University of Health Sciences, Karachi, PAK; 4 Cardiology, Lady Reading Hospital, Peshawar, PAK; 5 Internal Medicine, Kakatiya Medical College, Warangal, IND; 6 Anesthesiology, Mayo Clinic, Rochester, USA; 7 Medicine, Texas A&M University, College Station, USA; 8 Medicine, University of North Texas, Dallas, USA; 9 Internal Medicine, Pulmonary Associates, Corpus Christi, USA; 10 Clinical Medicine, University of Houston, Houston, USA

**Keywords:** left heart cardiac catheterization, axillary vein catheterization, right ventricular lead, artificial pacemaker, aberrant superior vena cava: anomalous large vessel, anomalous left superior vena cava

## Abstract

Persistent left superior vena cava (PLSVC) is a congenital venous anomaly. It is frequently associated with other cardiac anomalies. The presence of dual superior vena cava is due to the lack of development of the left cardinal vein in utero. The coronary sinus gets dilated as a result of increased blood flow to the right heart and may be seen on echocardiography. This case describes a 50-year-old lady who presented to the emergency department with lightheadedness, nausea, and vomiting for one day, and her electrocardiogram showed a heart rate of 30 beats per minute. A temporary pacemaker was placed. She had a history of asymptomatic PLSVC diagnosed six months ago through percutaneous coronary intervention. A permanent pacemaker was passed through PLSVC to access the right ventricle and she was discharged home after five days of an uneventful hospital course. Clinicians should be aware of this rare congenital anomaly and its potential complications, particularly in patients with unexplained syncope or bradycardia. Further research is needed to better comprehend the clinical presentation, diagnostic evaluation, and management of PLSVC-related cardiac abnormalities.

## Introduction

Persistent left superior vena cava (PLSVC) occurs because of the congenital anomalous presence of superior vena cava (SVC) on the left. Although the exact mechanism of PLSVC is unknown, it is hypothesized that the lack of development of the left cardinal vein in utero is responsible for the dual SVC [1]. PLSVC causes coronary sinus (CS) dilatation, which is one of the early findings detectable on echocardiography [2]. PLSVC is commonly found in patients with congenital heart disease (CHD). It is more common in patients with a ventricular septal defect and double outlet right ventricle disease than in other CHDs [3]. The overall prevalence of PLSVC ranges from 0.2% to 3% in the general healthy population and 11% in those with CHD [4]. PLSVC has been implicated in rhythm disorders and abnormal electrical axis. The electrical instability usually manifests as supraventricular arrhythmia with AV-nodal reentrant tachycardia (AVNRT) being the most common subtype [5]. This is the first-ever case report of PLSVC from South Asia, presenting with severe symptomatic sinus bradycardia and requiring a permanent pacemaker (PPM). This case highlights the importance of considering anatomical variations in the evaluation of bradycardia. This case report can serve as a reminder for clinicians to consider alternative etiologies for bradycardia in patients, including anatomic abnormalities. Additionally, this case report can serve as a starting point for further research into the prevalence of PLSVC and its association with bradycardia. By better understanding the incidence and significance of this anatomical variation, clinicians can improve their ability to diagnose and manage bradycardia in patients. Finally, the case report emphasizes the need for effective communication and collaboration between medical professionals involved in the care of a patient, including a cardiac electrophysiologist.

## Case presentation

A 50-year-old, stay-at-home lady, presented to the emergency department of a tertiary care hospital with light-headedness, nausea, and vomiting for one day. She was a known case of diabetes and hypertension for 10 years. She underwent a percutaneous coronary intervention (PCI) which showed blockage in the obtuse marginal (OM) branch of the left circumflex artery. The rest of the coronary vessels were normal. A stent was placed in the OM vessel six months ago. During the procedure, incidental asymptomatic PLSVC was found, and observation was advised.

**Figure 1:**
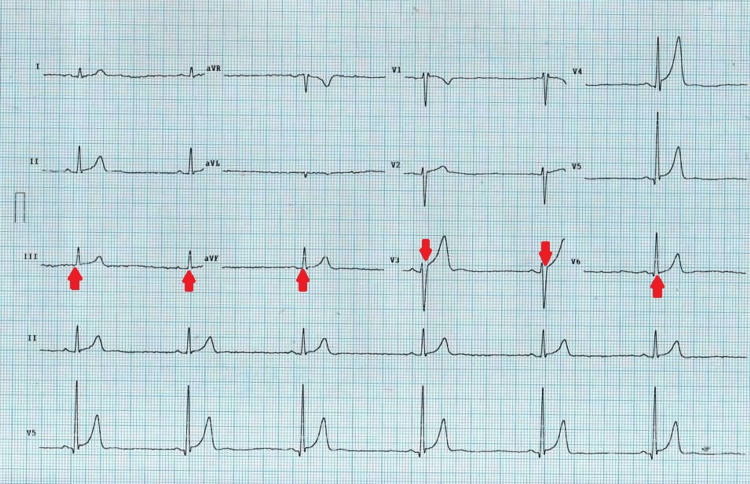
Electrocardiogram showing sinus bradycardia and a heart rate of 45 beats per minute.

On examination, she appeared sick but was oriented to time, place, and person. She was afebrile with a blood pressure of 130/80 mmHg, pulse rate of 40 beats per minute, and respiratory rate of 22 breaths per minute. An electrocardiogram showed sinus bradycardia (Figure 1). Baseline investigations and troponin levels were within the normal range. Echocardiography showed normal size and function of all chambers. The ejection fraction was found to be 45%. A temporary pacemaker was placed, and the patient was admitted to the cardiac unit.

**Figure 2:**
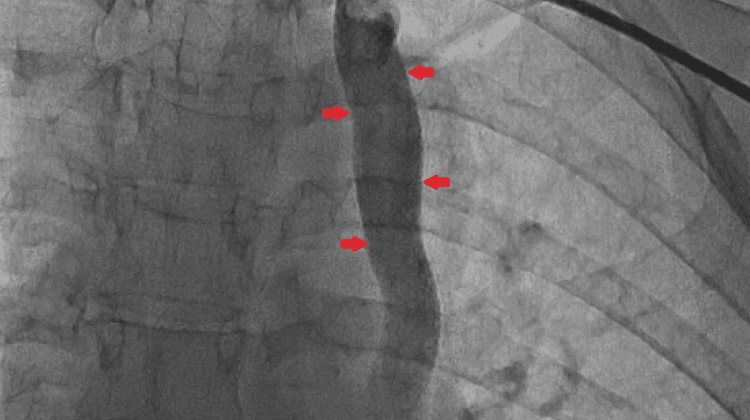
Fluoroscopy section: anteroposterior view showing superior vena cava marked with arrows.

**Figure 3:**
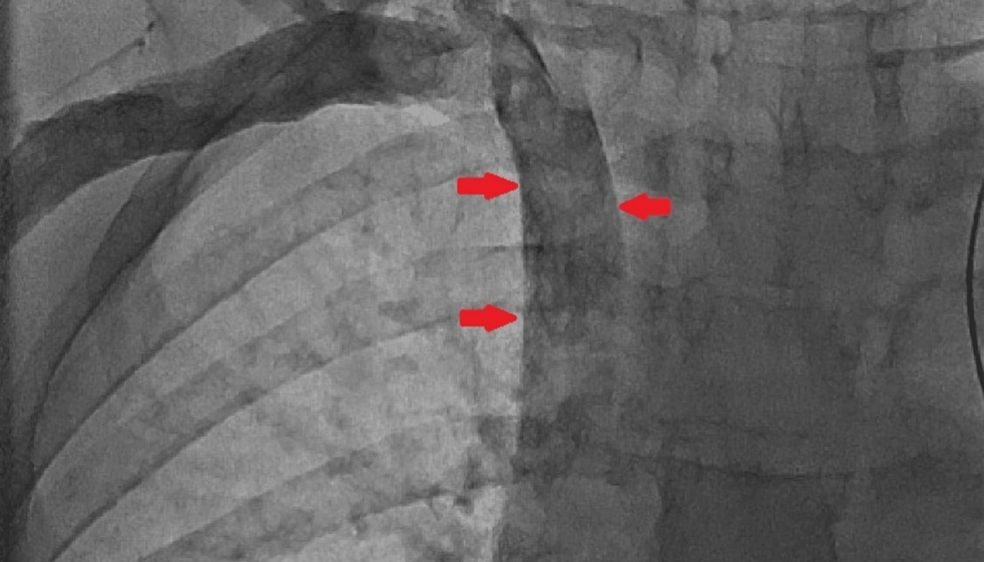
Fluoroscopy section: anteroposterior view showing persistent left superior vena cava marked by arrows.

Written informed consent was obtained for the placement of a PPM. Before the procedure, 600 mg of clopidogrel was administered orally in divided doses. Antiseptic measures were taken. Based on the previous PCI, the left axillary vein was approached. The guidewire was passed through the LPSVC, dilated CS, and the right atrium and then parked into the inferior vena cava (Figures 2, 3). A left subclavian pocket was created. The right ventricular (RV) lead was secured in the RV apex and the minimum threshold was set at 0.75 mV. The batteries were attached to the lead and sutures were placed between the skin and muscular layer to close the pocket. The patient had three days of postoperative stay in the hospital.

## Discussion

PLSVC is an uncommon congenital anomaly with various consequences for patients. PLSVC is known to be associated with complete heart block and pacemaker implantation in patients [5]. The existence of PLSVC has also been reported with coronary artery disease, although no causal link has been found [6]. Often, it is an asymptomatic condition that is diagnosed incidentally on imaging [6] or during pacemaker or catheter placement or cardiac surgery [7].

In this case, the patient presented with severe symptomatic sinus bradycardia due to PLSVC requiring PPM placement. Access to the RV was gained through the PLSVC. In patients requiring PPM, a PLSVC can lead to many complications. The methods used to overcome this setback include a hybrid approach with epicardial lead pacing, bi-atrial pacing, dual-chamber rate-adaptive pacemaker pacing with contralateral lead placement, or the typical RV lead placement, which was used in our case as well [5]. PLSVC increases the risk of sudden cardiac death, and the patient might be considered for corrective surgery [8].

PLSVC causes increased right atrial drainage, therefore, the CS is often enlarged. This enlargement can cause compression of the sino-atrioventricular node and bundle of His, leading to serious cardiac arrhythmia. During central venous catheter (CVC) insertion, cardiac resynchronization therapy, or pacemaker implantation, electrode fixation becomes challenging due to the tortuous course of PLSVC. CVC insertion without fluoroscopic guidance can cause angina, hypotension, and heart perforation. In the case of constricted CS, catheterization might lead to significant complications, such as arrhythmias, cardiogenic shock, and cardiac tamponade. Because of the steal effect of the hemiazygos venous system related to PLSVC, retrograde cardioplegia may fail despite PLSVC clamping [9]. The knowledge of PLSVC is critical during cardiopulmonary bypass to avoid problems related to surplus blood return and insufficient venous return to the pump [9].

## Conclusions

To conclude, PLSVC can present variable consequences. An enlarged CS on echocardiography requires confirmation with saline contrast for possible PLSVC. A high index of suspicion is needed whenever the guide wire inserted via the left subclavian vein takes a left-sided course. Besides technical difficulties associated with right heart access, additional risks related to PLSVC should be considered.
